# Exploration of treatment burden through examination of workload and patient capacity during transition onto kidney replacement therapy: a systematic review of qualitative research

**DOI:** 10.1186/s12916-025-03904-7

**Published:** 2025-02-04

**Authors:** Catrin Jones, Ross Cairns, Heather Walker, Silje Welsh, Benjamin Edgar, Karen Stevenson, Bhautesh D. Jani, Patrick B. Mark, David Kingsmore, Katie I. Gallacher

**Affiliations:** 1 School of Health and Wellbeing, University of Glasgow, Glasgow, Scotland; 2 NHS Lanarkshire, Bothwell, Scotland; 3 School of Cardiovascular and Metabolic Health, University of Glasgow, Glasgow, Scotland; 4 NHS Greater Glasgow and Clyde, Glasgow, Scotland

**Keywords:** Treatment burden, Patient capacity, Healthcare workload, Kidney replacement therapy, Haemodialysis, Peritoneal dialysis, Kidney transplant

## Abstract

**Background:**

Patients with advanced chronic kidney disease requiring initiation of kidney replacement therapy (KRT) are frequently asked to enact complex management plans. Treatment burden has been defined as the effect of healthcare workload and the capacity a person has to manage this workload has on wellbeing. The aim of this review is to examine the experience of healthcare workload and the factors that affect capacity to meet that workload for people transitioning onto KRT for the first time, using a framework synthesis of published literature informed by normalisation process theory (NPT) and theory of patient capacity (TPC).

**Methods:**

Medline, Scopus and CINAHL were systematically searched with manual citation and reference searching. Studies were included if meeting the criteria of adults aged 18 or over transitioning for the first time onto any modality of KRT (haemodialysis, peritoneal dialysis or kidney transplantation), using qualitative methodologies to describe any aspect of experiences of healthcare workload or any factors that affect capacity to manage workload were included. Abstracts and full papers were independently screened by two reviewers and data extraction and quality appraisal were also independently conducted by two reviewers. Qualitative data were analysed using framework synthesis informed by NPT and TPC.

**Results:**

A total of 24,380 studies were screened, 406 full texts were reviewed and 18 studies were included. There were four broad categories of workload described: making sense of KRT, working out what to do and how to do it, meeting the challenges of KRT, and reflecting on work done. Patient capacity influenced the experience of all types of workload and the treatment burden generated by the work.

**Conclusions:**

Transitioning onto KRT is a period of very high healthcare workload and potentially high treatment burden. The relationship between healthcare workload and capacity to handle workload is complex, multifactorial and changes over time. By better understanding workload, capacity and burden during transition, we can develop better ways of measuring these important aspects of care and develop interventions to reduce treatment burden in those transitioning onto KRT.

**Supplementary Information:**

The online version contains supplementary material available at 10.1186/s12916-025-03904-7.

## Background

### Conceptualising workload, capacity and treatment burden

For people with long-term conditions, there is considerable workload associated with following treatment regimens and managing health. In recent years, there has been interest in conceptualising healthcare workload and the subsequent impact it can have on wellbeing, defined as treatment burden [1]. Healthcare workload is the objective work that patients are asked to meet. Examples of healthcare workload include demands made upon the patient to organise and co-ordinate their own care, comply with complex treatment and self-monitoring regimes, and to meet numerous expectations of personal motivation, expertise and self-care [1]. The personal, physical, emotional, social, environmental and financial resources and abilities that a person can mobilise to meet the demands of their health management is defined as patient capacity [2, 3]. Patient capacity is not fixed, but a dynamic entity that can be augmented or diminished depending on the resources the patient has available to them [4]. Treatment burden is a product of the interaction between healthcare workload and patient capacity: it is the subjective experience of trying to realise healthcare workload and the resultant impact on wellbeing. This is closely related but different to ‘symptom burden’, which is the burden generated by the direct experience of symptoms related to the disease [1]. To understand and develop ways of minimising treatment burden, it is important to first understand the factors influencing healthcare workload and patient capacity, as interventions aimed at lessening workload or improving capacity have the potential to reduce burden [1].

Two middle-range theories have been used to describe how healthcare workload and capacity translate to everyday practice: normalisation process theory (NPT) and theory of patient capacity (TPC). NPT addresses workload: it identifies the work and processes that individuals and groups engage that promote and inhibit the routine incorporation of complex interventions into everyday life. It has four main components: coherence (sense-making work); cognitive participation (engagement work); collective action (operational work); and reflexive monitoring (appraisal work) [5]. NPT can be applied to understand how patients, their families and their wider social network meet healthcare workload, and how that impacts on their experiences of treatment burden [6–8]. The TPC addresses patient capacity as the product of the interaction of the five core domains: biography, resources, environment, realisation of work and social [4].

### Treatment burden during transition onto kidney replacement therapy

Chronic kidney disease (CKD) is defined as kidney dysfunction measured by estimated glomerular filtration rate (eGFR) lasting greater than 3 months as defined by the Kidney Disease Improving Global Outcomes staging. Kidney replacement therapy (KRT) is considered in the setting of severe kidney dysfunction, usually based on symptoms, clinical and biochemical manifestations of uraemia, and as a shared decision between the patient and nephrology team. Patients can elect to initiate KRT or choose comprehensive conservative care (CCM) [9]. KRT can be performed either by peritoneal dialysis (PD), haemodialysis (HD) or kidney transplantation (KTx) [10].

The period around the transition onto KRT from the point of identification of the imminent need for KRT to being fully established on maintenance therapy could be a time of particularly high treatment burden. Healthcare workload can include making key decisions regarding their care to enacting new complex self-care tasks such as attending the dialysis unit multiple times a week or undertaking home dialysis, managing new medication regimens and enacting dietary changes [10]. Adverse early dialysis experience can have a lasting impact on a patient’s treatment burden and overall patient journey [11].

However, previous research examining treatment burden, workload or patient capacity during transition onto KRT has been very limited. One synthesis studied the work of being a patient with chronic kidney disease but included a much broader population ranging from patients with mild CKD to those long established on KRT [12]. It demonstrated that CKD patients had a high treatment burden and that capacity to meet that burden is dynamic and multifactorial, but did not discuss the period of transition in detail. Another synthesis did study transition onto KRT, but analysed the data with themes based on lived experience and did not conceptualise in terms of treatment burden, workload or capacity [13].

The aim of this review is to explore the experience of healthcare workload and patient capacity for people transitioning onto KRT, through examination of the published literature by framework synthesis of published literature underpinned by NPT and TPC.

## Methods

The review protocol is registered on PROSPERO, the International Prospective Register of Systematic Reviews, registration number CRD42024513205. The methodology is reported in accordance with the ENTREQ (Enhancing Transparency in the Reporting the Synthesis of Qualitative Research) Statement [14] and the PRISMA (Preferred Reporting Items for Systematic Reviews and Meta-analyses) guidelines [15].

The inclusion and exclusion criteria described in accordance to the SPIDER tool [16] are shown in Table 1.

**Table 1:** Summary of inclusion and exclusion criteria

Sample	• Adults aged 18 or older transitioning onto any modality of KRT for the first time • Studies that included children, patients with earlier stages of CKD or that were established on maintenance KRT, or discussed transition between modalities were excluded • Studies that included caregivers or healthcare professionals were only included if reported in a way that allowed for the selective inclusion of direct patient experiences • Transition onto KRT is defined as the time period between identification of the imminent need for KRT and being fully established on maintenance therapy
Phenomenon of interest	• Patient experiences of any aspect of workload or treatment burden during transition onto KRT • Any descriptors of patient capacity or the effect of patient capacity on experiences
Design	• English language qualitative studies published in 2012 or later
Evaluation	• Direct patient experiences and perceptions
Research type	• Published peer-reviewed qualitative studies • Grey literature and quantitative methodologies including patient-reported measures were excluded • Studies of interventions were excluded

We used a comprehensive search strategy devised with input from an information scientist (Supplemental Material 1). Studies were limited to those published in 2012 or later to capture current practice. Due to the subject matter being poorly indexed, a broad inclusive approach was adopted to the search strategy. Medline, Scopus and CINAHL databases were searched with additional citation and reference tracking. Databases were initially searched in June 2023 and updated in February 2024. Following removal of duplicates, a two-stage screening process (title and abstract then full text) was conducted independently by two reviewers using DistillerSR software, with discrepancies resolved by a third reviewer.

Data were extracted independently by two reviewers for the year of publication, country, population, number of participants, KRT modality, data collection method, methodology and research question (Supplemental Material 2). Quality appraisal was conducted independently by two reviewers using the JBI Critical Appraisal Checklist for Qualitative Research [17], with discrepancies resolved by discussion with a third reviewer. No study was excluded on the basis of quality, and the quality appraisal did not affect the weighing given to the results of a particular study in the synthesis.

Qualitative analysis was conducted using NVivo 14. Both direct participant quotations and author’s descriptors and analysis in the result and discussion segments were included for analysis. We adopted an abductive approach to coding [18]. A coding framework was created informed by the domains and sub-domains of normalisation process theory (coherence, cognitive participation, collective action, reflective monitoring) to describe workload [19] and the theory of patient capacity (biography, resources, environment, realisation of work and social functioning) to describe capacity [4] with any phenomena that fell outside these theories coded independently (Supplemental Material 3). One researcher coded all the papers, and 6/18 were double coded by a second researcher to ensure consistency of coding. The coding frame was refined iteratively.

Line by line coding was conducted to search for concepts, and comparisons were made within and across studies using a framework synthesis approach. Themes were derived abductively: initially there was a deductive derivation from the framework synthesis then subsequently an abductive higher order process of synthesising new findings in order to conceptualise treatment burden during transition onto KRT. This generated a taxonomy of treatment burden grounded in primary research but underpinned by NPT and TPC.

## Results

**Fig. 1:**
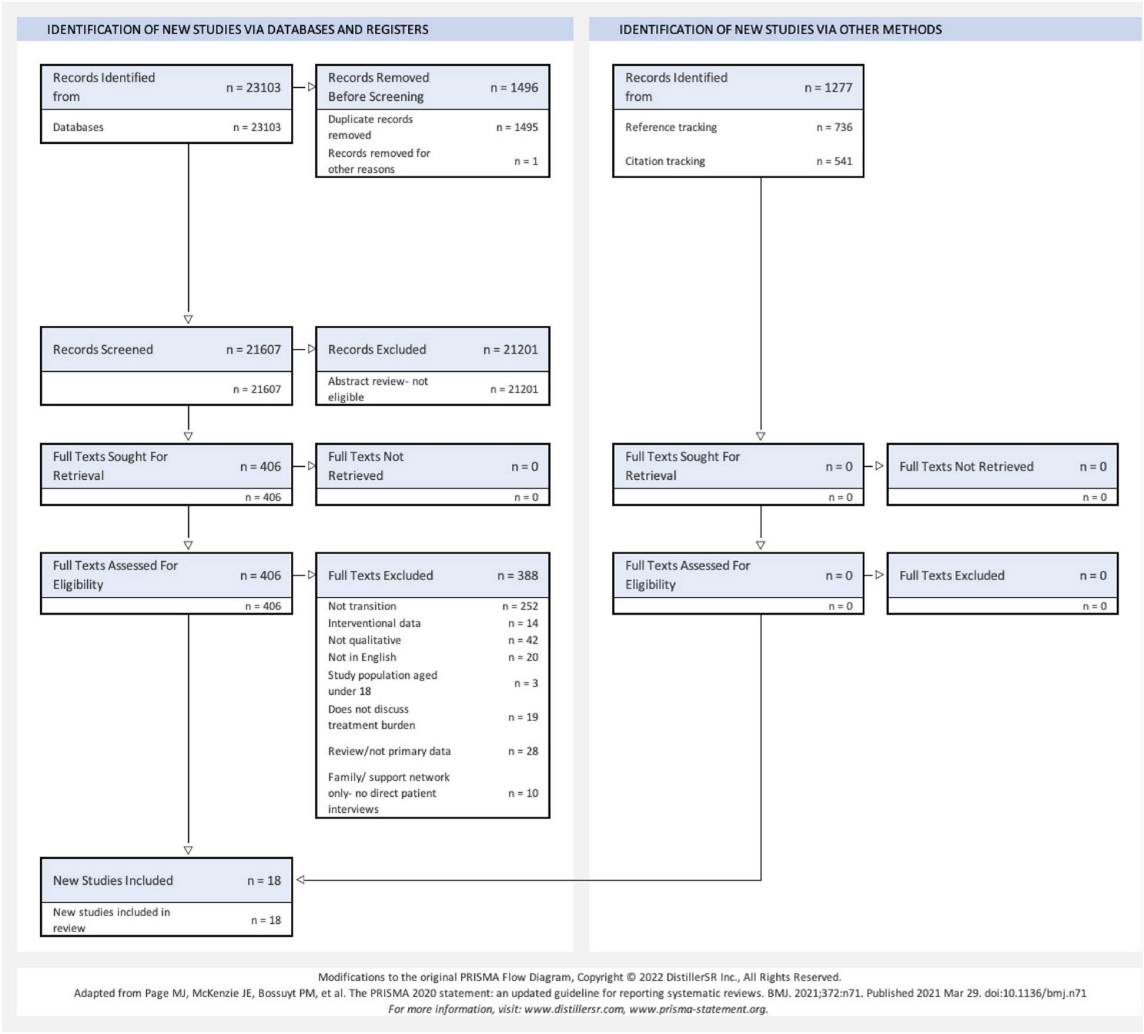
Flow diagram demonstrating identification process for eligible papers. Legend: Modified PRISMA flow diagram demonstrating the identification and selection process for eligible papers. Databases screened were Medline, Scopus and CINAHL

A total of 18 studies were included for analysis [20–37] (see Fig. 1). The included studies were from a wide range of countries: Taiwan [21, 24, 34], New Zealand [22, 27, 31], Australia [32, 35], Singapore [23, 28], Denmark [29, 37], USA [25, 30], Canada [36], Iran [20], UK [33] and Sweden [26]. Participant numbers in the included studies ranged from 5 to 168. Eight studies had a haemodialysis only patient population sample, 1 had only peritoneal dialysis patients in their sample and 9 studies had a mixed sample. Out of the 18 included studies, 17 used key informant interviews and 1 used focus group discussions. A total of 16 studies interviewed their participants once; 2 had serial interviews. The included studies are summarised in Table 2. The studies were of mostly moderate or high quality [17]. The quality assessments of the included studies are summarised in Table 3.

**Table 2:** Description of included papers

Author	Year	Country	Participants	Population	KRT	Methodology	Data collection	Serial interviews	Study aim
Alsing	2022	Denmark	8	Patients that have recently started in centre HD	HD	Thematic analysis	FGD	No	To explore expectations and experiences of patients who have recently started HD
Cassidy	2018	Canada	12	4 patients on in-centre HD, 4 on home HD and 4 on PD in Ontario	HD PD	Content analysis	KII	No	To understand the dialysis modality decision-making process
Erlang	2015	Denmark	9	Patients that have undergone a pre-dialysis educational programme	HD PD KTx	Phenomenology	KII	No	To explore patient experiences of involvement in the dialysis modality decision-making process
Griva	2019	Singapore	97	People approaching dialysis or very recently started HD	HD	Thematic analysis	KII	No	To explore perspectives of various stakeholders on barriers to timely dialysis access creation
Gullick	2016	Australia	11	People who had commenced haemodialysis within the previous 3 months at a public hospital in Sydney	HD	Phenomenology	KII	No	To interpret the spatio-temporal experience of people with kidney failure and their families in the first months of haemodialysis
Hassani	2017	Iran	24	People starting HD in Iran	HD	Grounded theory	KII	No	To explore the process of transition to haemodialysis
Henry	2017	USA	168	Patients initiating HD at Kaiser Permanente Southern California	HD	Thematic analysis	KII	No	To characterise the experiences of patients starting KRT
Ho	2021	Taiwan	31	Patients with a established diagnosis of CKD in the process of decision-making regarding dialysis	HD PD KTx	Thematic analysis	KII	Yes	To describe the psychological change process of CKD patients during the shared decision-making process
Ho	2022	Taiwan	15	Patients who had been through a hospital based shared decision-making process before starting PD	PD	Thematic analysis	KII	No	To explore the process of information acquisition and consideration during shared decision-making for patients undergoing PD and compare their quality of life and expectations before and after PD at home
Lai	2012	Singapore	13	Patients starting haemodialysis using a CVC in Singapore. Age 39–63. 7 females, 6 men. Chinese and Malay speaking	HD	Phenomenology	KII	No	To identify main concerns and needs encountered by incident haemodialysis patients during the early months on haemodialysis
Lin	2022	Taiwan	5	Patients who required emergency HD starts	HD	Content analysis	KII	No	To explore experiences of urgent dialysis patients
Monaro	2014	Australia	11	Patients within 3 months of HD start in a tertiary referral hospital in Sydney	HD	Phenomenology	KII	No	To explore early experiences of close family members and people with kidney failure commencing HD in a tertiary hospital
Nilsson	2019	Sweden	5	Patients who have experienced emergency HD starts with CVC	HD	Phenomenology	KII	No	To explore experiences of starting dialysis in an unplanned fashion
Novick	2021	USA	15	Spanish speaking patients reliant on emergency-only dialysis (no health insurance)	HD	Thematic analysis	KII	No	To understand the kidney disease educational gaps of Latinx individuals who need but lack access to scheduled outpatient dialysis
Walker	2016a	New Zealand	52	Mixed European, Maori and Pacific Islander population that are either immediately pre-dialysis or stated within last 12 months	HD PD	Thematic analysis	KII	No	To describe patient and caregiver values, beliefs and experiences when considering home dialysis
Walker	2016b	New Zealand	52	Mixed European, Maori and Pacific Islander population that are either immediately pre-dialysis or stated within last 12 months	HD PD	Thematic analysis	KII	No	To describe patient and caregiver perspective of the economic considerations that influence dialysis modality choice
Walker	2017	New Zealand	13	Maori patients who had received education about treatment modalities or started dialysis within the last 12 months	HD PD	Thematic analysis	KII	No	To explore and describe Maori patient’s experience and perspectives of chronic kidney disease
Winterbottom	2012	UK	20	Attending low clearance clinic in a large hospital in northern England. 90% White; 10% Asian	HD PD	Thematic analysis	KII	Yes	To understand how patients with kidney failure make dialysis treatment decisions

*KII* Key informant interview, *FGD* Focus group discussion

**Table 3:** Quality assessment of included papers (JBI Critical Assessment Tool for Qualitative Research) [14]

Author	Congruity between philosophical perspective and methodology	Congruity between methodology and research question	Congruity between methodology and methods used to collect data	Congruity between methodology and data representation and analysis	Congruity between methodology and interpretation of the results	Cultural or theoretical position of researcher	Influence of researcher on research discussed	Adequate representation of the participants and their voices	Ethical approval	Conclusions flow from the data analysis	Subjective global assessment
Alsing	Yes	Yes	Yes	Yes	Yes	Yes	Yes	Yes	Yes	Yes	High quality
Cassidy	Yes	Yes	Yes	Yes	Yes	No	No	Yes	Yes	Yes	Moderate quality
Erlang	Yes	Yes	Yes	Yes	Yes	No	No	Yes	Yes	Yes	Moderate quality
Griva	Yes	Yes	Yes	Yes	Yes	No	No	Yes	Yes	Yes	Moderate quality
Gullick	Yes	Yes	Yes	Yes	Yes	No	No	Yes	Yes	Yes	Moderate quality
Hassani	Unclear	Yes	Yes	Unclear	Unclear	No	No	Yes	Yes	No	Low quality
Henry	No	Unclear	Yes	Yes	Yes	No	No	Yes	Yes	Yes	Moderate quality
Ho (2021)	Yes	Yes	Yes	Yes	Yes	No	No	Yes	Yes	Yes	Moderate quality
Ho (2022)	Yes	Yes	Yes	Yes	Yes	No	No	Yes	Yes	Yes	Moderate quality
Lai	Yes	Yes	Yes	Yes	Yes	No	No	Yes	Yes	Yes	Moderate quality
Lin	Unclear	Yes	Yes	Yes	Yes	No	No	No	Yes	Unclear	Low quality
Morano	Yes	Yes	Yes	Yes	Yes	Yes	Yes	Yes	Yes	Yes	High quality
Nilsson	Yes	Yes	Yes	Yes	Yes	Yes	Yes	Yes	Yes	Yes	High quality
Novick	Yes	Yes	Yes	Yes	Yes	Yes	Yes	Yes	Yes	Yes	High quality
Walker (2016a)	Yes	Yes	Yes	Yes	Yes	Yes	No	Yes	Yes	Yes	High quality
Walker (2016b)	Yes	Yes	Yes	Yes	Yes	No	No	Yes	Yes	Yes	Moderate quality
Walker (2017)	Yes	Yes	Yes	Yes	Yes	Yes	Yes	Yes	Yes	Yes	High quality
Winterbottom	Yes	Yes	Yes	Yes	Yes	No	No	Yes	Yes	Yes	Moderate quality

There were 3059 items coded. Only 45 items fell outside the NPT and TPC derived framework and required separate coding, demonstrating their suitability for conceptualising treatment burden during transition onto KRT. These items were participants’ recommendations for change and specific discussions of trauma.

### Workload

There were four broad categories of work described: making sense of KRT; working out what to do, and how to do it; meeting the challenges of KRT; and reflecting on work done (Table 4). These correspond to the four main components of NPT: coherence, cognitive participation, collective action and reflexive monitoring [19] but we have adapted the terminology to be more specific to KRT transition.

**Table 4:** Workload associated with KRT initiation identified from the literature: a taxonomy using NPT

NPT category	Taxonomy
**Making sense of KRT (*Coherence Building*)**	
Working out how and why life on KRT will be different (*Differentiation*)	Shock and alarm at sudden transition from being asymptomatic to being very ill, denial and attempts to avoid dealing with imminent need for KRT for as long as possible
	Challenges of understanding and accepting lifelong requirement for treatment
	Grieving a lost life and the loss of future hopes and dreams
	Fears of premature death
	Anger at missed opportunities to diagnose and optimise
Understanding what KRT means for social network (*Communal specification*)	Understanding the emotional, physical, psychological and financial burden placed on family and friends meeting the workload of KRT
	Making decisions collectively for the wider benefit of the family unit
	Impact of new dependency of family role, e.g. as a parent, grandparent or spouse
	Impact of potential for live donor transplantation on family relationships
	Impact of shame and stigma of being ill and needing KRT on their ability to socialise
Understanding what KRT will mean for them as an individual (*Individual specification*)	Key considerations with KRT decisions: • Ability to maintain employment • Location of dialysis unit and availability of transport • Available support from family and healthcare staff • Dietary changes • Ability to travel abroad, continue hobbies • Attitude to procedures: need for operation, changes in appearance, dislike of needles • Preservation of independence and social role • Confidence in personal capacity • Time commitment • Financial implications and out of pocket costs
	Accessing and interpreting official resources (leaflets, workshops, clinical consultations and specialist nurse support) to understand their options
	Accessing and interpreting informal/alternative sources of information
	Navigating conflicting advice
	Visiting dialysis unit to see what haemodialysis involved
Finding value in KRT (*Internalisation*)	Understanding how a future life on KRT can still be fulfilling, and how adaptations can be made to preserve elements of current lifestyle
	Worsening physical symptoms demonstrating need for KRT for quality of life and survival
	Hope of a future transplant: seeing the work created by PD/HD as a temporising measure until kidney transplantation and the hope of greater normality
	Positive and negative influence of peers and family on their expectations of life on KRT
	Seeking alternative therapies, e.g. traditional Chinese medicine
	Empowerment from being supported to make their own decisions about KRT modality
**Working out what to do, and how to do it (*Cognitive Participation*)**	
Driving forward work required to start KRT (*Initiation*)	Adapting living arrangements to facilitate KRT: carrying out home modifications, buying new furniture, moving to new accommodation closer to dialysis centre
	Financial adaptation: changing or leaving employment, financial planning, having to sell assets including homes if unable to afford mortgage, navigating benefit system
	Attending clinics and having procedures such as creation of an arteriovenous fistula or insertion of a peritoneal dialysis catheter in preparation to start
	Emergency/unexpected KRT starts required to do all the work of starting and the work of making sense of KRT simultaneously increasing strain and feelings of disempowerment
	Overcoming anxiety about starting
	Adapting dietary and fluid consumption habits
Getting others to join in (*Enrolment*)	Recruiting friends and family to assist with activities of daily living and transport; ability of social network to meet this workload key determinant of viability of some options
	Using peers and family members as a source of knowledge and decision-making support
	Pressure of KRT destroying some relationships
	Enrolling support from the wider kidney patient community
	Complex dynamics of living donation of kidney: both from seeking donation and managing emotional and psychosocial effects of donation offers from family
Deciding that the chosen type of KRT is the right thing to do (*Legitimisation*)	Finding out that there is better outcomes associated with their choices (e.g. home vs in-centre dialysis, AVF vs CVC) increased confidence that they had made the right choices and any additional work was worth it
	Improvement in physical symptoms and energy levels legitimising decision to start
	Understanding consequences of complications validates their preventative efforts
	Maintaining jobs, family and community roles increased confidence in treatment choices
	Unexpected out of pocket costs (e.g. higher electricity bills with home dialysis) and financial hardship undermining confidence in system/choices
	Ongoing fatigue and distressing physical symptoms such as itch undermined confidence
Maintaining key interventions in daily life (*Activation*)	Ability to maintain social connectedness: context dependent with in-centre dialysis allowing regular socialising with fellow patients important for some, whilst home based therapies facilitating better continuity of pre-existing social connections for others
	Successfully navigating first sessions increases confidence in manageability of life on KRT
	Reassurance and motivation in seeing others successfully establish on KRT and do well
	Pre-dialysis education left patients well equipped to manage by themselves once started
**Working with their social network and the healthcare system to meet the challenges of KRT (*Collective Action*)**	
Doing work required by KRT (*Interactional workability*)	Home therapies (both PD and HD) came with significantly increased workload but minimised the disruption caused to people’s lives and therefore facilitated them maintaining employment and consequent financial security and their family/social role
	CAPD patients need to find suitable clean places to change the dialysate during the day, which poses significant restrictions on
	Difficulty coordinating work necessitated by KRT with other responsibilities in life
	Making the most of free time in between dialysis days to retain some pleasure in life
	Organising transportation
	Maintaining dietary and fluid restrictions in daily life
Effect on relationships and the confidence people have in each other (*Relational integration*)	Changing relationship dynamics from one where they were equals or provided for others to one where they are dependent and require care psychologically difficult for all involved
	Familial love and support major source of sustenance
	Fatigue, geographical limitations and time taken up by KRT making socialising difficult
	Fatigue and time taken up by KRT reducing available time to fulfil family role, e.g. parent
	Not wanting others to see them unwell and ‘weak’ leading to social withdrawal
	Financial hardship and pressure on spouse to shoulder most household work and additional care responsibilities putting significant pressure on household relationships
	Effect of involvement in decision-making and preparation: families that had been involved through the process felt engage, families that had been excluded felt disconnected increasing the amount of isolation perceived by the patient
Appropriateness of the allocation of work (*Skillset workability*)	Lack of support to access benefits that the patients are eligible leaving patients with impossible choices between employment and health
	Provision of online resources unsuitable for those unable to access internet
	Appropriate support of additional work leading to patients feeling empowered
Effect of interaction with healthcare or governmental services on patients (*Contextual integration*)	Previous poor experiences of healthcare and poor health outcomes (potentially resulting in need for KRT) undermining confidence in future ability to manage their health and confidence in clinical team
	Fears that clinicians had an agenda to push a particular form of KRT and they were not given a balanced choice
	Fears that clinicians had made incorrect assumptions about their ability to cope with certain modalities of KRT, mistrust that their options had been unfairly curtailed
	Being given information in ways that did not meet their needs: shame and stigma of having to disclose poor literacy, non-fluency in dominant regional language (e.g. English), poor digital literacy and lack of access to the internet and/or visual impairment
	Information presented in ways that are too fast and complex, and significant power imbalance leading to them not being able to understand information given or ask for the information in other ways
	Trusting and reciprocated relationship with clinicians enhancing ability to ask questions, participate in shared decision-making and have confidence in decisions made
	Poor relationship with clinicians, feeling that they are not being listened to or respected undermining trust in system
	Comfort in having confidence in the skill and expertise of their clinical team and feeling that they are well cared for currently and in future
	Clinicians respecting and recognising cultural context and working to find treatment solutions that works with and respects important things for patient
	Insufficient resources provided for different cultural needs, e.g. no dietary advice for cuisines other than dominant majority regional culture
	Healthcare system facilitating and supporting peer-to-peer support such as with ‘Kidney Schools’
	Poor information flow with patients needing to be proactive and advocate for themselves in consultations to get the required information out of their clinical team
	Significant amount of work and costs transferred from healthcare system to patients and their families with home-based therapies with insufficient reimbursement or support
	Difficulty navigating multiple government agencies and accessing welfare grants and benefits. Obtuse and obstructive processes, people feeling worn down by being continually required to justify their need for assistance
	Lack of health insurance, citizenship status or private financial means severely curtailing care on offer
	Healthcare professionals providing excellent information about the technical and physical side of KRT but neglecting the emotional aspects
	Poor employment rights with employers not mandated to make adjustments to facilitate KRT or pay sick leave
**Reflecting and evaluating on work done (*Reflexive Monitoring*)**	
4.1 Finding information about the effects of KRT and its components (*Systematisation*)	Talking with other patients about their experiences
	Searching for information online
4.2 Evaluating effect of KRT on family/social network (*Communal appraisal*)	Ability to maintain role and responsibilities within family key motivating factor for persevering with difficult/intrusive treatments
	Working out together how to adapt their lives to make time for family
	Benefits seen from keeping their loved one alive and well outweigh burden of care
	Increasing experience increasing the family’s confidence in their ability to meet the work
4.3 Evaluating the effect of KRT on themselves (*Individual appraisal*)	Evaluating change: those who were highly symptomatic prior to starting saw a greater improvement in symptoms and therefore greater value in the treatment whereas those with a lower symptom burden and more active lifestyle more affected by disruption
	Worsening physical health despite KRT undermined confidence in the process
	Life on KRT was substantially better than what most patients’ feared prior to starting
	Conflicting emotions with gratitude on one hand that they are still alive and receiving good care, and sadness and grief for their previous life on the other
4.4 Adapting as a result of reflections (*Reconfiguration*)	Learning how to let go of any previous understandings of constructively spent time and accepting time spent on dialysis: learning to stop thinking of it as wasted or lost time
	Normalising KRT in their daily lives and finding ways of reintroducing hobbies
	Finding activities that they can do to entertain and distract during dialysis sessions
	Learning how to continuously evaluate what it possible on a daily basis and adapt
	Finding flexible jobs that can accommodate their needs on KRT

#### Making sense of KRT

The work of making sense of KRT was commonly reported, including understanding how life could be different on KRT, understanding what KRT means for them as a patient, their family and wider social network, and finding value in KRT.

The work involved in psychological preparation and overcoming profound fears was a common theme [21, 23, 26, 27, 30, 34, 35].

*Many patients were concerned about either the surgical access procedure, dialysis itself or both. Fear of pain/discomfort, needles, seeing one’s own blood and changes to their physical appearance; all of these were repeatedly mentioned as concerns driving ambivalence towards preparation and initiation of RRT* [30].

The difficulty of first understanding their disease and then communicating what that means to their family, as well as needing to explain their social context to their medical team and working out how best to proceed in a complex and multifactorial situation was a recurring theme [22–24, 26, 27, 29, 31, 34–37].

*“It’s really hard to explain sometimes that family are first, that I am not an individual, that I am part of a unit, that then no decision is just mine, but it’s also really hard to explain to my whanau [extended family group] what is happening with my kidneys when I don’t really know it so well myself”* [27].

Patients were required to understand complex treatment options and contextualise them to their own lives and work out what was desirable and feasible for them [17–26, 28, 30–34].

*“The fact that I live by myself and I am really not medically inclined at all, made me choose [IC-HD]…I just didn’t feel comfortable to be able to do it myself at home. If I had a partner, it would have been different…I think I would have gone for the peritoneal”* [36].

#### Working out what to do, and how to do it

Studies reported a considerable workload associated with preparing for transition. This included driving forward the work required to start KRT, enrolling the support of others, determining that the chosen modality of KRT is the right thing to do and maintaining key interventions in daily life.

The impact of transitioning onto KRT was far-reaching, with patients required to enact a wide range of adaptations from changing or leaving employment, financial planning, adapting their diets and overhauling their weekly schedule to accommodate dialysis sessions [21, 22, 25, 26, 29–37].

*Because I’ve put my house up for sale, erm, because otherwise I’m not going to be able to afford to pay the mortgage or anything. I’ve had to make that decision* [33].

Home dialysis patients had to make considerable adaptations to their home environment [22, 27, 31, 32, 36].

*Garry had to compartmentalise space in his home into a no-go zone, making ‘home time and space’ into ‘treatment time and space’: “We had to make changes in the house…barricade our bedroom now so that Nicholas [infant son] can get used to the fact that he can’t go in that room”* [32].

Experiencing new dependency on others and the need to enrol friends and family to assist with both material and emotional support was a common finding in all studies [20–37]. The potential tensions caused by the possibility of live donor transplantation were also explored:

*The issue of living organ donation further complicated family dynamics. Some participants were reluctant to ask family to consider organ donation. Others were particular about whom they would approach* [35].

#### Meeting the challenges of KRT

Studies describe patients working with their social network and healthcare system to meet the challenges of KRT. This includes doing the work that is required, the effect it has on relationships and the confidence people have in each other, the appropriateness of the allocation of work and the effects of interaction with the healthcare and other governmental systems.

Many different types of work were reported, from attending hospital appointments, meeting dietary restrictions, attending dialysis sessions, arranging transport, taking medication, carrying out tasks relating to home dialysis such as exchanging dialysate, and adapting plans to accommodate the requirements of KRT [20–37].

*Many participants described the need to coordinate their lives to match the efforts and challenges of replacing the dialysate (…) their lives and work must be changed to accommodate the dialysis time* [24].

The work of KRT caused people’s relationships to change, and often required families and friends to undertake considerable caring work to assist the person undergoing KRT [22, 24, 26–28, 31, 32, 35–37].

*Long-term management may mean families undertake a range of complex activities such as active facilitation of home-based HD. Families and friends often assist with activities of daily living and transport. This level of involvement places considerable pressure on personal relationship and may incur feelings of anxiety, depression and isolation from other relatives and friends* [35].

The relationship with their healthcare team was identified as an important factor in patient’s experience in many studies [20–22, 24–31, 33, 36, 37].

*Nephrologists play a significant role in modality education and decision making. When a trusting partnership was established, patients had an enhanced sense of importance, control and respect. When patients felt like valued members of the HCT, they were more likely to be receptive to information, be engaged in their care, and participate in shared decision making* [36].

Transitioning onto KRT often required patients to interact with governmental and social welfare systems which are bureaucratic and involve significant effort to access support [22, 24].

*Participants struggled to access financial support both from their dialysis service and government agencies and described difficulty in navigating the social welfare system. Many felt disempowered by the system, and worn down by the need to continually justify their requirements for assistance* [22].

#### Reflecting on work done

Studies report the work involved in finding information about the effect of KRT and its components, evaluating the effect of KRT on the family and social network, evaluating the effect of KRT on themselves and adapting as a result of reflections.

Reflecting on experiences of dialysis and adapting and reframing expectations of life was a key part of reflexive monitoring in this cohort [20, 22, 24, 26, 29–32, 36].

*Dealing with ‘clock time’ meant letting go of any previous understanding of constructively lived time and accepting a different construct of being and time on the dialysis machine. Sharon filled her time in a measured way. She described time-filling and pacing as a learned skill* [32].

Building on previous experiences and learning new self-management skills lead to greater confidence over time in meeting the work of KRT [24, 26, 32, 37].

*Learning to deal with previous hardships gave some strength and tools to accept and handle the difficult new situation. When dialysis finally became regular it became easier to feel more self-assured. The new routine gradually gave back structure to life* [26].

### Patient capacity

The five factors that were reported as affecting capacity to manage health were biography; resources; environment; realisation of work; and social functioning (Table 5).

**Table 5:** Patient capacity during transition onto KRT identified in the literature: taxonomy using TPC

Theory of patient capacity category	Taxonomy
**Biography**	
**Biographical disruption**	
Fear of the future and fear of death	Fears of mortality: understanding the permanent and life-threatening nature of kidney failure, the need for KRT to preserve life and fears of dying despite dialysis
	Fear of procedures: pain, needles, alteration of physical appearance, blood
	Fears of losing quality of life: fear of suffering, fear of decline in health and capacity
Loss of meaningful life	Being tied to the rigid timetable if dialysis- losing spontaneity and independence
	Losing social connectedness as socialising more difficult as a result of the physical symptoms of kidney failure and the restrictions from KRT
	Losing their identity and sense of self
Loss of future hopes and dreams	Uncertainty making it difficult to plan and have aspirations and goals
	Difficulty planning and partaking in enjoyable things such as holidays
Loss of role	Losing role within family especially transitioning from someone who cared/provided for others to someone dependent on others
	Needing to retire due to ill health and give up job (and associated identity)
	Stigma of ill health, men feeling emasculated by requiring care
**Biographical adaptation**	
Reframing future expectations	Looking forward to symptom reduction on dialysis
	Framing PD/HD work as a positive to keep them alive until kidney transplantation
	Accepting the resulting change in relationship dynamics from new dependency
Finding a new role in life	Finding ways to adapt and continue important life activities
**Resources**	
**Financial**	Financial loss as a result of reduced working hours/ unplanned early retirement
	Needing to sell assets including homes if unable to meet previous financial commitments
	Need to meet unexpected out of pocket expenses: increased electricity bills with home dialysis, home adaptations, travel costs to hospital visits
	Ability to continue work and minimise financial hardship key in treatment decisions
	Insurance status affecting treatment options
	Poverty and poor quality housing limiting options (e.g. home dialysis)
**Literacy**	Ability to understand and communicate in the dominant regional language
	Ability to read and write (especially visual impairment)
	Ability to read long and complex information and understand medical terminology
	Digital literacy—ability to access and navigate online resources
**Medical knowledge**	Empowerment to engage in shared decision-making gained from knowledge about their disease and prognosis
	Understanding their disease prior to progression to kidney failure allowed for better adjustment and preparation
	Knowledge gained from talking to other patients and peers
	Gap in information given: no focus on emotional/psychological factors
	Disempowerment and distress from not having adequate medical knowledge
**Paid support services**	Home visits support from specialist nurses to facilitate home dialysis
**Physical health**	Dealing with severe fatigue and fluctuating energy levels
	Difficult symptoms: itch, swelling, cramp, breathlessness, nausea, thirst, weakness
	Feeling too unwell to engage in discussions or take on and interpret information
	Insomnia and disruption to sleep patterns
	Changing physical appearance: scarring and disfigurement from vascular access procedures, weight gain from medication
	Improving health once established on KRT
	Managing other co-morbidities and their symptoms
**Psychological resilience**	Ability to accept and process their diagnosis and prognosis
	Ability to stay hopeful and find pleasure in life
	Ability to retain self-identity and self-worth as health deteriorate, overcome stigma
	Ability to overcome feelings of fear and/or despair
	Ability to overcome anger and regret at missed opportunities to intervene earlier
**Self-efficacy**	Confidence in their ability to undertake KRT related tasks
	Ability to self-advocate in discussions with healthcare staff
	Confidence in their ability to navigate healthcare services
	Confidence in their ability to negotiate with other key stakeholders such as employers/governmental agencies to access resources and support
**Time**	Time to prepare prior to starting KRT allowing planning, education and pre-emptive access creation
	Time involved in centre dialysis: whole week dependent of treatment timetable, losing autonomy over their own time
	Rigidity of treatment timetables heavily constraining ability to make time for other things: family, friends, work
	Negotiating treatment timings in order to be able to meet other responsibilities
**Transport**	Needing to depend on family/friends for lifts to hospital
	Personal ability to drive
	Challenges with accessing hospital transport
**Environment**	
**Capacity building environments**	Reliable access to clinical advice and support enhanced confidence
	Culturally competent care, with care being available in patients’ native language and advice adapted for the cultural context it is being given
	Actions of employers in allowing more flexible working around KRT requirements
	Comprehensive patient education
**Person centred care**	Clinicians actively trying to engage with patients and enable them to develop trust and regain power and confidence in their decision-making
	Being cared for in ways that accommodates and realises the importance of community, cultural and spiritual connections
	Clinicians making time to listen to patients and discuss concerns
**Negative experiences**	Feeling unable to discuss issues such as financial hardship due to stigma
	Feeling forced and coerced into treatment decisions
	Staff being too busy and overworked to spend time supporting patients
	Being overloaded with information and decisions when too unwell to process
**Realisation of work**	
**Enhancing capacity**	Work realised for home dialysis enhancing capacity by minimising disruption
	Increasing confidence by successful execution of tasks
**Reducing capacity**	Being overwhelmed by work and not coping diminishes confidence and capacity
**Social functioning**	
**Ability of their social network to accept condition and effects**	Strain on relationships from new dependency; perception of non-reciprocity in some relationships exacerbating feelings of burden
	Reciprocity, constancy and love in spousal relationship
	Ability of social network to understand the diagnosis and prognosis
	Ability of friends and family to adapt relationship to new constraints
	Desire to protect family and friends from the disease exacerbating isolation
**Personal ability to fulfil social role**	Losing ability to socialise due to fatigue and intrusiveness of the strict schedule and time commitments required on KRT
	Depression exacerbating social withdrawal
	Ability to continue in job, family and community roles key to sense of identity and self-worth; profound impact if lost
	New social connections made with other patients during KRT
**Provision of instrumental support**	Ability of family members or friends to act as kidney donor and ability of potential recipient to accept donation
	Ability of social network to assist with activities of daily living and transport
	Ability of social network to provide emotional support
	Ability of friends and family to discuss options and help with decision-making
	Need for adaptations at work in order to be able to continue working
	Provision of peer-to-peer support from other KRT patients
**Social relationship with their healthcare team**	Patient-physician partnership crucial to shared decision-making: mutual trust key to engagement, sense of control and ability participate in shared decision-making
	Informal and formal support from dialysis nursing team
	Anger and mistrust in healthcare team from missed opportunities to diagnose and treat kidney disease earlier, potentially avoiding need for KRT
	Shame, embarrassment and unequal power dynamics leading to non-disclosure of factors such as poverty and illiteracy to healthcare team
	Trust lost if patients feel that doctors have an agenda to push one modality or did not fully explain and explore options
	Confidence gained by demonstration of competence and compassion

#### Biography

Profound biographical disruption, meaning disruption to a person’s ability to perform their usual roles, was a common finding [20–23, 25–32, 34, 35, 37]. KRT could disrupt biography which in turn affects capacity to manage healthcare workload.

The challenge of confronting their own mortality in the context of a lifelong, life-threatening illness was a common finding, with many studies describing participants’ intense fear of the future and fear of death [20, 23, 26–28, 30, 32, 35, 37].

*It was the fear of the unknown. I thought I was going to be invalid for the rest of my life and… I was saying “No Mum, I’m going to die”* [35].

Distress from losing their previous social role either within the family or in the wider community and dealing with new dependency was a common finding, with many reporting shame, stigma and social withdrawal [20, 23, 26, 27, 30, 31, 34, 35, 37].

*Many participants, often men, associated sickness with weakness and inferiority from their peers. For men who had always been physically active and perceived as strong, the need to be dependent on others and a machine made them feel ashamed and often led to withdrawing from family and not participating in dialysis education and preparation* [27].

KRT limiting people’s ability to socialise and carry out their normal daily routine was common [20–28, 30–35, 37].

*Intrusiveness of haemodialysis on preferred lifestyle and activities emerged as patients’ major concerns. Limitations of work, travel, social life as well as fluid and diet restrictions were discussed and linked to feelings of despair and frustration* [23].

Biographical adaptation, or reframing, is when patients find ways to reframe their expectations of life and find meaning in their lives with a chronic illness [4]. This process where patients find meaning in their new lives was described in some studies [20, 23, 26, 35, 37].

*Participants mentioned haemodialysis acceptance with time and insight improvement. Normalizing haemodialysis, living in dialysis ward, maintaining prior self-image, enduring haemodialysis and coping with it, complying life activities with haemodialysis and considering the dialysis machine as part of the body* [20].

#### Resources

Studies reported the importance of accessing and mobilising critical resources. Financial resources were commonly discussed [20–26, 28, 30–36]. Some people had already retired and were financially comfortable with reliable affordable access to healthcare which enhanced their capacity to manage health. However, KRT can disrupt ability to work and the resultant financial hardship from reduction or loss of income was profound for many. Some chose to pursue home KRT in order to be able to continue working; however, the directly incurred costs of home KRT could be considerable due to the necessary home modifications and additional power and water consumption. Patients’ ability to meet these costs was a major determinant of the viability of some therapeutic options.

*She was afraid of the machine using lots of power. She was worrying it would be too expensive to run it (…) ‘We have such a tight budget now, to add anything even five dollars of power, that could tip us over, so that meant the machine at home was out’* [31].

Other directly incurred costs also created hardship such as fuel costs to drive to hospital appointments, especially if rural. In countries without universal healthcare, the cost of insurance or paying for treatments could be problematic, with some relying on emergency only HD if routine care was unaffordable [20, 21, 25].

Literacy and prior medical knowledge were important determinants of capacity. Patients who were able to easily access and understand information were better prepared to transition onto KRT and fully engage in shared decision-making [21, 23, 24, 27–31, 33–36]. Access to information and support from peers or family members with prior experience of KRT could be a valuable resource [24, 26, 27, 30, 31, 33]. Patients with limited knowledge of kidney disease or poor health literacy found the medical terminology and the volume of new information overwhelming. Patients who did not speak the dominant regional language (e.g. English), came from a different culture to the dominant regional culture, were visually impaired or had poor digital literacy were further disadvantaged [25, 27, 31].

*Participants experienced confusion during discussions with clinicians. Their limited understanding of kidney disease made it difficult to process what they were told, ask questions, and make decisions. The large volume of information conveyed at one time made it especially difficult to process what was happening. Most participants believed they were in kidney failure because of their nutritional habits, but did not demonstrate a clear understanding of what caused their kidney disease or how it could have been prevented* [25].

Physical health and physical abilities were key resources. Distressing symptoms such as fatigue, vertigo and muscle cramps limited capacity to cope with new treatment and maintain social connection [23, 25, 26, 30, 32, 33, 35, 37]. Patients who had to start dialysis as an emergency were often critically ill and were too unwell to process the information being given to them or participate in shared decision-making [25, 26].

*Participants reported they often felt too ill to engage in discussions with clinicians. Since most education happened while they were experiencing symptoms of volume overload and uraemia, the felt too sick to understand, inquire about options or make decisions*. *They reported being only interested in feeling better, not in understanding what is happening to their bodies, and would have accepted any treatment option without resistance* [25].

However, as patients felt better on dialysis and had fewer symptoms their capacity to engage with KRT was enhanced.

Psychological resilience was an important determinant of capacity. Many studies described participants who felt that their sense of identity and self-worth had been severely undermined by illness and new dependency. Depression, withdrawal and suicidal thoughts were commonly described [21, 23, 25–27, 30, 35–37].

*For some male participants, their gendered construct of identity was severely challenged, and in Peter’s case, this led him to feelings of worthlessness: “A lot of blokes would suffer depression…like I felt..Feeling useless, that they’re no good for anybody…I’ve always been independent felt that it was a man’s place to support his family. Ever since I’ve been sick I’ve been relying on the family to keep me going..F-ing useless! I should have died [crying] and it would have been better for everybody ‘cause it’s so hard on everyone now”* [35].

Feelings of guilt, anger or regret at missed opportunities for earlier intervention could undermine patients’ confidence in their ability to self-care and healthcare professionals’ ability to care for them leading to reduced capacity [25, 27, 28, 30, 31, 37].

*Many participants, particularly those with diabetes, expressed regret that they could have avoided or delayed dialysis. Despite many acknowledging they had not known enough to make significant changes earlier, many blamed themselves for not proactively asking about treatment or lifestyle changes, or trying to understand more about their condition to help them self-manage their care, internalising a sense of inadequacy. These experiences often led to loss of confidence in their own ability to care for themselves* [27].

Conversely, those who were able to adjust, remain optimistic and find value and legitimacy in KRT were better equipped to meet their workload. Confidence in ability to undertake KRT related work, ability to navigate complex systems and self-advocate in interactions with healthcare professionals and other agencies influenced capacity [20, 27, 28, 31, 33, 34, 36]. Self-efficacy was especially important when engaging in shared decision-making around treatment choices.

*“I’m embarrassed to say, it’s actually a lot of education to learn it [home dialysis], I have to learn how to do the machine, and they say it’s hard, and it takes a long time, I guess I’m just not sure if I can learn it, and I’m not that good, and I felt a lot of pressure to learn at their level and I didn’t really understand, but I don’t want to tell them or they’ll think I’m dumb”* [27].

Time to prepare for transition to KRT was important: patients who had been aware of the likely need for KRT for months had had time to prepare practically and psychologically, and key components of an optimal start such as pre-emptive vascular access, consideration of live donor transplantation and pre-dialysis education could be enacted [22, 36]. Patients who needed to start dialysis urgently did not have this preparation time which undermined their capacity [21, 26, 35–37].

*The abrupt onset of treatment appeared to allow little time for expectations, increasing the initial shock. Emotional displays during the discussions gave the impression of not having come to terms with one’s current life situation* [37].

The effect of time spent on KRT was also important, with some finding the time commitment and rigid schedules of KRT intrusive and overwhelming, taking them away from other aspects of their life that gave them self-worth and purpose and therefore capacity [20–24, 26–28, 30–33, 35–37].

*“Haemodialysis is three times per week and I have to wait (at the dialysis centre). Time for work becomes a problem…and time to spend with my family”* [23].

Available transport affected capacity, especially for patients who lived rurally and needed to travel great distances. This sometimes resulted in them needing to move to live close to the hospital, disrupting their support network and so diminishing their capacity [22, 27, 31, 32, 37].

#### Environment

Patients had their capacity enhanced by a person centred environment where they felt respected, understood and cared for and had easy access to support and suitable resources [26, 27, 29–31, 34, 36]. Cultural competence was an important theme in studies that captured the experiences of minoritised communities. Being able to access care in their native language and access advice adapted to their culture (e.g. dietary guidance) enhanced capacity [25, 31]. Equally for Māori patients feeling that clinicians understood and respected the importance of the spiritual connection to land and people and the importance of taking that into account when making treatment decisions enhanced their capacity [27]. Conversely, indifferent or stigmatising care or failure to meet key needs diminished capacity [22, 24–27, 29, 31, 36].

*Participants who were illiterate or for whom English was a second language felt lost and embarrassed about being unable to read and understand the information given. As such, they indicated to clinicians that they understood the information* [31].

*Despite the need of guidance, some of the participants pointed out that lack of time and the busyness of staff often obstructed the possibility of engaging in dialogue. Furthermore, it seemed necessary for patients to take an active and questioning approach in the consultation at the outpatient clinic beyond just basic questions* [29].

#### Realisation of work

Capacity affects an individual’s ability to realise work, but conversely the realisation of work has an impact on capacity: sometimes carrying out workload successfully enhanced capacity and reduced burden [4]. The effect of this relationship was clear in papers discussing home dialysis [22, 24, 27, 31]. Home HD or PD has considerable additional workload compared to in-centre HD and therefore requires significant capacity for it to be an option. However, there is a paradoxical relationship where the emotional and cognitive reinforcement from undertaking the work required to dialyse at home and the resultant multifactorial enhancement in capacity from being able to continue employment, maintain family and community roles, and minimise life disruption results in the increased workload reducing overall burden compared with in centre dialysis.

*Being able to dialyse when it was convenient for them allowed participants to maintain financial security and themselves and their family; this was particularly important if they were the sole provider* [31].

#### Social functioning

Patients who were able to adapt and maintain their social roles in the workplace, community or family gained confidence in the process and described features of enhanced capacity [22, 27, 28, 31]. However, for others a loss of social role was felt profoundly and could further diminish capacity due to loss of social connectedness and biographical disruption [20–28, 32, 35, 37].

*For some the need of dialysis meant not being able to travel or return to work. This was a real disappointment. Dependence on dialysis also affected life at home as roles were forced to change. Some participants found their inability to contribute as much as before as very frustrating, leading to feelings of guilt. “It has become so that my wife has to do everything. Before I cooked food and fixed things and now she has to do it all…yes so I feel it’s my fault”* [26].

In many studies, patients reported receiving wide ranging support from their family and community; emotional support, assistance with activities of daily living and transport, and decision-making support [27–29, 33–36]. Those without much of a social network lacked this support. For some, new dependency led to concerns about the viability of their relationships and sometimes resulted in marital difficulty and relationship breakdown [23, 27–29, 32, 35].

*Vanessa lived with her brother who provided her with no practical help. Asking for assistance from people other than family placed dependence in a different dimension for Vanessa, “I’ve got to now ask for help from my friends…I’m finding it difficult to do so”* [35].

The social relationship between patients and healthcare professionals influenced capacity. A trusting and mutually respectful relationship enhanced capacity [20, 24, 26–31, 33, 34, 36, 37]. However dysfunctional patient-clinician relationships severely impaired capacity and profoundly increased the sense-making and cognitive participation workload encountered by patients [24, 25, 27–31, 34, 36].

*During clinical consultations to discuss modality preferences, some felt powerless to articulate their concerns particularly if they perceived that their doctor was ‘sitting up on a ladder’ talking to them. They felt unable to question or believe that they were expected to immediately comprehend and understand the information regarding each modality. Some participants felt so disempowered during clinical encounters that they instinctively chose the safest option, facility dialysis* [31].

## Discussion

**Fig. 2:**
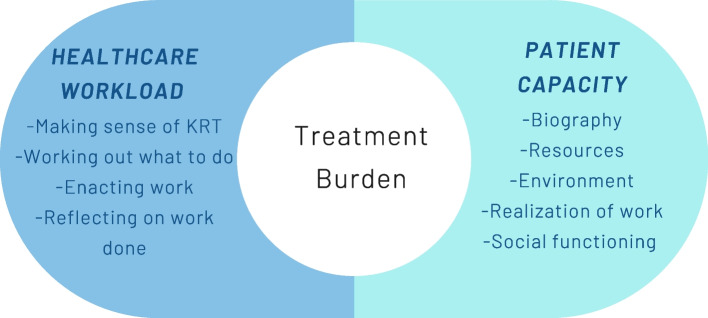
Demonstration of the relationship between healthcare workload, patient capacity and treatment burden

Treatment burden is a product of the interaction between healthcare workload and capacity (Fig. 2). This systematic review describes a considerable healthcare workload for people transitioning onto KRT and many important factors that affect capacity to manage that workload. None of the included papers comprehensively discussed all aspects of workload and capacity across the whole patient journey but all papers captured a part of the patient experience of the transition process. Therefore, when synthesised together in this review, a broader exploration of treatment burden during transition onto KRT is provided. By developing a taxonomy of workload and a taxonomy of capacity, we can describe the different components that influence a patient’s experience of overall treatment burden.

### Comparison with treatment burden in other diseases

Our findings align with previous work on treatment burden that demonstrated the relationship between workload and patient capacity is complex, multifactorial, temporally dynamic and dependent on personal circumstance. It cannot be characterised as a simple inversely proportional relationship between workload and capacity [38]. Understanding the treatment burden encountered by patients involves understanding the interaction between the totality of the multitude of different workloads they are required to meet and the range of different domains that contribute to their capacity: some enhancing it and some diminishing [38].

A systematic review looking at treatment burden across a range of long-term conditions conceptualised three common spheres of treatment burden: biographical disruption encompassing the loss of freedom and independence and restriction of meaningful activities, relational disruption encompassing social isolation and relationship strain, and biological disruption encompassing the physical side effects [39]. This fits closely with the findings of this review, emphasising the universality of treatment burden as a concept and the common features that manifest across a wide range of life experiences and diseases.

However, experiences of treatment burden described in this review did differ from studies that have examined other conditions in some aspects. In KRT transition the magnitude and intensity of the required healthcare contact involved in in-centre dialysis, the complexity of the tasks needed to be carried out to facilitate home dialysis and the intrusion of KRT related activity into daily life differed from that described for other conditions such as congestive heart failure [6], stroke [40], cancer [41] and type 2 diabetes [42]. The focus of different components of workload was also different, with greater emphasis on understanding concepts and working out what to do in the KRT transition population compared to congestive heart failure [6]. The effect on families and the wider social network differed in particular, partially because the KRT population captured a wider range of ages and included younger working age patients with young families as well as older patients. Their described workload and capacity factors were centred around managing a life-threatening disease with young families and maintaining ongoing employment more prominently than in studies of heart failure [6] and stroke [40], but was similar to that described in cancer [41]. Polypharmacy and medication workload was a more prominent feature of treatment burden in congestive heart failure [6], type 2 diabetes [42] and stroke [40] than in KRT.

### Comparison to other studies of effect of patient capacity on transition onto KRT

A study that used a combination of patient-reported measures to quantify the relationship between different domains of capacity and illness intrusiveness on haemodialysis found that reduced physical, mental and financial capacity were robustly correlated with increased patient-reported disruption [43]. Although this study had a long-term maintenance haemodialysis only population, their findings correlate with the findings of this review.

Patient activation is a different but overlapping concept to patient capacity. Patient activation has an individualistic focus on a patient’s own knowledge, belief, motivation, confidence and skills in managing a chronic disease [44] and puts the onus on the patient to become ‘activated’ in order to become a more efficient manager of their own health needs [45] whereas patient capacity conceptualises a patient’s ability to meet healthcare workload more holistically as a synergy of individual, social and environmental factors [4]. Studies of patient activation in kidney disease have found that lower activation is associated with higher symptom burden and lower quality of life [46], and that activation scores are higher in those starting home dialysis and those who had pre-dialysis nephrology care [47]. This correlates with qualitative descriptions of patient capacity in this review.

### Strengths, limitations and future research needs

The strength of this review lies in its tight focus on transition and its exhaustive search, which allowed a detailed qualitative exploration.

Our inclusion criteria were limited to English language publications published in 2012 or later. The rationale for the temporal limitation was to capture current practice. Although our review included papers a wide variety of countries, the English-language limitation may have excluded relevant papers from other countries. Papers were mostly published from highly developed countries, and there was a paucity of data from low and middle-income countries with no included papers from either Africa or South America.

We limited the inclusion criteria to studies of patients experiencing initial transition onto KRT and excluded papers that included experiences of patients on long-term maintenance KRT, transitioning between modalities and those who chose conservative care, which limited the scope of the review. The benefit of this is that it facilitated a focused and comprehensive discussion of treatment burden during KRT transition which has different patterns of workload and burden compared to experiences of CKD more generally described elsewhere [12]. However, a consequence of this is that only experiences of transitioning onto KRT by way of pre-emptive kidney transplantation were captured, rather than the more common scenario of a period of dialysis with subsequent transplantation. More research is required to explore the treatment burden of transition pathways to kidney transplantation.

Treatment burden is dynamic and changes over time, and as most of the included papers only interviewed their participants once, the evolution of an individual’s treatment burden over the process of transition was not captured in this review and is an area that would benefit from further research.

This review describes the different workloads encountered and the different factors that underpin capacity but further qualitative research is required to gain a deeper understanding of the dynamic relationship between patient capacity and workload during transition. Specifically, more work is required to understand the complex interplay between different types of workload encountered and different patient capacity factors such as the extent of the biographical disruption encountered and ability to reframe and enact biographical adaptation; the financial, literacy, medical knowledge, physical health, psychological resilience, self-efficacy and time resources utilised; the individual’s relationship with their social network and the ability of that network to support them; and the wider environment in which patients are trying to meet this workload and how this evolves over time.

We encountered less discussion than anticipated on the effect of multiple long-term conditions (multimorbidity) and managing other conditions whilst transitioning onto KRT, especially considering that the prevalence of multimorbidity in the CKD5 population is 97.5% and the prevalence of complex multimorbidity defined as 4 or more long-term conditions is 66.2% [48]. More work is required to explore this aspect.

## Conclusions

This review is a comprehensive examination of healthcare workload and capacity to manage that workload during patient’s first transition onto KRT. This emphasises the importance of taking treatment burden into account when caring for patients preparing to transition onto KRT.

Through further research to better understand workload, capacity and burden during transition, we can develop better ways of measuring burden and recognising patients at risk of becoming overburdened, and develop interventions to reduce workload, enhance capacity and reduce treatment burden, which can potentially improve care experiences for these patients.

## Supplementary Information

Supplementary Material 1.

Supplementary Material 2.

Supplementary Material 3.

## Data Availability

The data that support the findings of this study are available from the corresponding author upon reasonable request. All requests for data access should be directed to the corresponding author. The search strategy and coding framework are supplied as supplementary materials.

## Abbreviations

KRT: Kidney replacement therapy
NPT: Normalisation process theory
TPC: Theory of patient capacity
CKD: Chronic kidney disease
eGFR: Estimated glomerular filtration rate
CCM: Comprehensive conservative management
PD: Peritoneal dialysis
HD: Haemodialysis
KTx: Kidney transplant
ENTREQ: Enhancing Transparency in the Reporting the Synthesis of Qualitative Research
PRISMA: Preferred Reporting Items for Systematic Reviews and Meta-analyses
