# Supplementary material for: Exploration of treatment burden through examination of workload and patient capacity during transition onto kidney replacement therapy: a systematic review of qualitative research
Source: BMC Med. 2025 Feb 4;23:61. doi: 10.1186/s12916-025-03904-7 (PMC11792302; doi:10.1186/s12916-025-03904-7)
Supplement: Supplementary file 1 — Supplementary Material 1. [file 12916_2025_3904_MOESM1_ESM.docx]

**Database: Ovid MEDLINE(R) ALL <1946 to May 10, 2023>**
**Search Strategy:**
**1**  Renal Replacement Therapy/ (6872)
**2**  ((renal or kidney) adj replacement therapy).tw. (17015)
**3**  ((kidney or renal or pre?emptive) adj transplant*).tw. (87626)
**4**  exp Renal Dialysis/ (125686)
**5**  dialysis.tw. (120584)
**6**  (hemodialysis or haemodialysis).tw. (86642)
**7**  (endstage kidney or endstage renal or end stage kidney or end stage renal).tw. (49690)
**8**  (ESKD or ESKF or ESRD or ESRF).tw. (21845)
**9**  (kidney adj failure).tw. (9828)
**10**  (renal adj (insufficiency or care)).tw. (23722)
**11**  (hemofiltration or haemofiltration).tw. (4614)
**12**  ultrafiltration.tw. (17666)
**13**  vascular access.tw. (11277)
**14**  peritoneal dialysis.tw. (28030)
**15**  or/1-14 (341440)
**16**  ((Treatment* or (disease* adj3 management) or interven* or medicat* or therap* or medicin* or (patient* adj3 care) or selfcare or self-care or (health adj3 program$)) adj3 workload*).tw. (574)
**17**  ((Treatment* or (disease* adj3 management) or interven* or medicat* or therap* or medicin* or (patient* adj3 care) or selfcare or self-care or (health adj3 program$)) adj3 impact*).tw. (59056)
**18**  ((drug* or treat* or illness or procedure? or interven* or medicat* or therap* or medicin* or (patient* adj3 care) or selfcare or self-care or (health adj3 program*)) adj3 burden*).tw. (14994)
**19**  exp Quality of Life/ (265284)
**20**  exp Patient-Centered Care/ (23997)
**21**  (minimally disruptive medicine or patient-cent* care or chronic care or lived experience? or psycho?social experience? or decisions or BoT or QoL or HRQoL or TB).tw. (345478)
**22**  or/16-21 (634335)
**23**  Workload/ (23844)
**24**  Self-Management/ (5268)
**25**  disease management/ (42964)
**26**  Work/ (20245)
**27**  (work or workload or utili?ation review or process assess* or analys* or efficien* or task performance or task analys* or health?care contacts or health?care events or self-management or disease management or training or complex* or CuCoM).tw. (9507147)
**28**  or/23-27 (9555324)
**29**  coping.mp. or physician-patient relations/ or communicat*.mp. or partnership.mp. or socioeconomic*.mp. or psychosocial*.mp. or attitude*.mp. or acceptance of health care/ or acceptance.mp. or denial.mp. or choice*.mp. or resilien*.mp. or mobiliz*.mp. or mobilis*.mp. or "social conflict*".mp. or "physical status".mp. or "physical function*".mp. (2050821)
**30**  ("quality of life" or "well-being" or dysfunction* or community* or work or intimacy or intimate or emotion* or mood* or unmet or preference* or participat* or "side effect*" or emotion*).mp. (4273067)
**31**  (multimorbid* or comorbid* or morbidities).mp. (333483)
**32**  (multiple adj3 (chronic or illness* or morbid* or disease* or condition*)).mp. (50801)
**33**  ((multi or co) adj morbid*).mp. (34153)
**34**  "more chronic".mp. (3896)
**35**  or/29-34 (5913229)
**36**  Qualitative Research/ (80936)
**37**  Nursing Methodology Research/ (16407)
**38**  Questionnaires/ (559486)
**39**  exp Attitude/ (634914)
**40**  Focus Groups/ (35613)
**41**  discourse analysis.mp. (2512)
**42**  content analysis.mp. (42299)
**43**  ethnographic research.mp. (1230)
**44**  ethnological research.mp. (8)
**45**  ethnonursing research.mp. (42)
**46**  constant comparative method.mp. (1928)
**47**  qualitative validity.mp. (23)
**48**  purposive sample.mp. (4547)
**49**  observational method$.mp. (976)
**50**  field stud$.mp. (18168)
**51**  theoretical sampl$.mp. (909)
**52**  phenomenology/ (0)
**53**  phenomenological research.mp. (669)
**54**  life experience$.mp. (7532)
**55**  cluster sampl$.mp. (9432)
**56**  ethnonursing.af. (127)
**57**  ethnograph$.mp. (13823)
**58**  phenomenol$.af. (33855)
**59**  grounded theory.mp. (15101)
**60**  (grounded adj (theor$ or study or studies or research or analys?s)).af. (15333)
**61**  (life stor$ or women's stor$).af. (1702)
**62**  (emic or etic or hermeneutic$ or heuristic$ or semiotic$).af. or (data adj1 saturat$).tw. or participant observ$.tw. (30223)
**63**  (social construct$ or (postmodern$ or post-structural$) or (post structural$ or poststructural$) or post modern$ or post-modern$ or feminis$ or interpret$).mp. (619938)
**64**  (action research or cooperative inquir$ or co operative inquir$ or co-operative inquir$).mp. (5641)
**65**  (humanistic or existential or experiential or paradigm$).mp. (192076)
**66**  (field adj (study or studies or research)).tw. (19508)
**67**  human science.tw. (265)
**68**  biographical method.tw. (21)
**69**  qualitative validity.af. (23)
**70**  purposive sampl$.af. (12054)
**71**  theoretical sampl$.af. (909)
**72**  ((purpos$ adj4 sampl$) or (focus adj group$)).af. (89137)
**73**  (account or accounts or unstructured or open-ended or open ended or text$ or narrative$).mp. (811101)
**74**  (life world or life-world or conversation analys?s or personal experience$ or theoretical saturation).mp. (17744)
**75**  lived experience$.tw. (10855)
**76**  life experience$.mp. (7532)
**77**  cluster sampl$.mp. (9432)
**78**  (theme$ or thematic).mp. (165879)
**79**  categor$.mp. (474419)
**80**  observational method$.af. (977)
**81**  field stud$.mp. (18168)
**82**  focus group$.af. (69038)
**83**  questionnaire$.mp. (940099)
**84**  content analysis.af. (42318)
**85**  thematic analysis.af. (37874)
**86**  constant comparative.af. (3915)
**87**  discourse analys?s.af. (2559)
**88**  ((discourse$ or discurs$) adj3 analys?s).tw. (2982)
**89**  (constant adj (comparative or comparison)).af. (5950)
**90**  narrative analys?s.af. (1854)
**91**  heidegger$.tw. (769)
**92**  colaizzi$.tw. (1118)
**93**  speigelberg$.tw. (2)
**94**  (van adj manen$).tw. (529)
**95**  (van adj kaam$).tw. (44)
**96**  (merleau adj ponty$).tw. (260)
**97**  husserl$.tw. (308)
**98**  giorgi$.tw. (858)
**99**  foucault$.tw. (953)
**100**  (corbin$ adj2 strauss$).tw. (439)
**101**  (strauss$ adj2 corbin$).tw. (439)
**102**  (glaser$ adj2 strauss$).tw. (119)
**103**  glaser$.tw. (1084)
**104**  findings.af. (2561355)
**105**  interview$.af. or Interviews/ (483317)
**106**  qualitative.af. (340078)
**107**  or/36-106 (5767911)
**108**  22 or 28 or 35 (12733572)
**109**  15 and 107 and 108 (30202)
**110**  " 'Beats the alternative but it messes up your life': aboriginal people's experience of haemodialysis in rural Australia ".ti. (1)
**111**  " African American kidney transplant patients' perspectives on challenges in the living donation process ".ti. (1)
**112**  " Compartmentalising time and space: a phenomenological interpretation of the temporal experience of commencing haemodialysis ".ti. (1)
**113**  " Dialysis modality decision-making for older adults with chronic kidney disease ".ti. (1)
**114**  " Engagement in decision-making and patient satisfaction: a qualitative study of older patients' perceptions of dialysis initiation and modality decisions ".ti. (1)
**115**  " Experiences of patients undergoing dialysis who are from ethnic and racial minorities ".ti. (1)
**116**  " The perspectives of Aboriginal patients and their health care providers on improving the quality of hemodialysis services: a qualitative study ".ti. (1)
**117**  " Patient perspectives on the optimal start of renal replacement therapy ".ti. (1)
**118**  " Patient and caregiver values, beliefs and experiences when considering home dialysis as a treatment option: a semi-structured interview study ".ti. (1)
**119**  " Patient experience after kidney transplant: a conceptual framework of treatment burden ".ti. (1)
**120**  " Patient experiences of training and transition to home haemodialysis: A mixed-methods study ".ti. (1)
**121**  " You know your own fistula, it becomes a part of you --Patient perspectives on vascular access: A semistructured interview study ".ti. (1)
**122**  " The psychosocial experience of patients with end-stage renal disease and its impact on quality of life: findings from a needs assessment to shape a service ".ti. (1)
**123**  " Waiting for a kidney transplant: the experience of patients with end-stage renal disease in South Korea ".ti. (1)
**124**  " Working to establish 'normality' post-transplant: a qualitative study of kidney transplant patients ".ti. (1)
**125**  or/110-124 (15)
**126**  109 and 125 (15)
**127**  limit 109 to yr="2012 -Current" (18115)
