# Supplementary material for: Exploration of treatment burden through examination of workload and patient capacity during transition onto kidney replacement therapy: a systematic review of qualitative research
Source: BMC Med. 2025 Feb 4;23:61. doi: 10.1186/s12916-025-03904-7 (PMC11792302; doi:10.1186/s12916-025-03904-7)
Supplement: Supplementary file 2 — Supplementary Material 2. [file 12916_2025_3904_MOESM2_ESM.docx]

**Data Extraction Form**

1. Title
2. Journal
3. Authors
4. Year of Publication
5. Country
6. Number of participants
7. Description of population
8. Date of data collection
9. Healthcare funding
   1. Government funded/Universal healthcare
   2. Medicaid/ State based Insurance/ Co-pay
   3. Private/ Self funded
   4. Mixed
   5. Other
   6. Not stated
10. Number of sites:
    1. 1
    2. 2-3
    3. 4-5
    4. 5+
    5. Not stated
11. KRT modality:
    1. PD
    2. HD
    3. KTx
    4. Mixed
12. What methodological orientation was stated to underpin the study?
    1. Grounded theory
    2. Discourse analysis
    3. Ethnography
    4. Phenomenology
    5. Content analysis
    6. Other
13. How were patients selected?
    1. Purposive
    2. Convenience
    3. Consecutive
    4. Snowball
    5. Other
    6. Not stated
14. How was the data collected?
    1. Key Informant Interviews
    2. Focus groups
    3. Observation
    4. Mixed
    5. Others
15. Were repeat interviews carried out?
    1. Yes
    2. No
16. What aspects of transition were covered?
    1. Decision making on modality
    2. Experiences of procedures
    3. Physical adaptation
    4. Lifestyle adaptation
    5. Emotional adaptation
    6. Biographical disruption
    7. Impact on family/social life
    8. Financial/economic
    9. Other

**Joanna Briggs Quality Screen**

1. Is there congruity between the stated philosophical perspective and the research methodology?
   1. Yes
   2. No
   3. Unclear
   4. Not applicable
2. Is there congruity between the research methodology and the research question or objectives?
   1. Yes
   2. No
   3. Unclear
   4. Not applicable
3. Is there congruity between the research methodology and the methods used to collect data?
   1. Yes
   2. No
   3. Unclear
   4. Not applicable
4. Is there congruity between the research methodology and the representation and analysis of data?
   1. Yes
   2. No
   3. Unclear
   4. Not applicable
5. Is there congruity between the research methodology and the interpretation of results?
   1. Yes
   2. No
   3. Unclear
   4. Not applicable
6. Is there a statement locating the researcher culturally or theoretically?
   1. Yes
   2. No
   3. Unclear
   4. Not applicable
7. Is the influence of the researcher on the research, and vice versa, addressed?
   1. Yes
   2. No
   3. Unclear
   4. Not applicable
8. Are participants and their voices adequately represented?
   1. Yes
   2. No
   3. Unclear
   4. Not applicable
9. Is the research ethical according to current criteria or is there evidence of ethical approval by an appropriate body?
   1. Yes
   2. No
   3. Unclear
   4. Not applicable
10. Do the conclusions drawn in the research report flow from the analysis or interpretation of the data?
    1. Yes
    2. No
    3. Unclear
    4. Not applicable

Subjective global assessment

1. As a whole, how would you appraise the quality of this study?
   1. High quality
   2. Mostly high quality but some aspects lower quality/absent
   3. Low quality
2. Additional comments
