# Supplementary material for: Exploration of treatment burden through examination of workload and patient capacity during transition onto kidney replacement therapy: a systematic review of qualitative research
Source: BMC Med. 2025 Feb 4;23:61. doi: 10.1186/s12916-025-03904-7 (PMC11792302; doi:10.1186/s12916-025-03904-7)
Supplement: Supplementary file 3 — Supplementary Material 3. [file 12916_2025_3904_MOESM3_ESM.docx]

**Coding Framework**

**Workload**

Coherence Building

- - Differentiation
  - Communal Specification
  - Individual Specification
  - Internalisation

Cognitive Participation

- - Initiation
  - Enrolment
  - Legitimisation
  - Activation

Collective Action

- Interactional Workability
- Relational Integration
- Skillset Workability
- Contextual Integration

Reflexive Monitoring

- Systematisation
- Communal Appraisal
- Individual Appraisal
- Reconfiguration

**Capacity**

Biography

- Biographical adaptation
  - Finding a new role in life
  - Reframing future expectations
- Biographical disruption
  - Fear of future suffering
  - Loss of a meaningful life
  - Loss of future dreams, hopes and aspirations
  - Loss of role

Resources

- Financial
- Literacy
- Medical knowledge
- Paid support services
- Physical abilities
- Physical energy
- Physical health
- Psychological resilience
- Self-efficacy
- Time
- Transport

Environment

- Capacity building environment
- Experiences of person-centred care
- Negative experiences

Realisation of Work

- Realisation of work affecting capacity

Social Functioning

- Ability of their social network to accept condition and effects
- Personal ability to socialise
- Provision of instrumental support
- Social relationship with their healthcare teams

**Other**

- Cultural differences
- Experiences of procedures
- Recommendations for improvement
- Trauma
